# Multicenter validation of the flow measurement of classical monocyte fraction for chronic myelomonocytic leukemia diagnosis

**DOI:** 10.1038/s41408-018-0146-8

**Published:** 2018-11-14

**Authors:** Sihem Tarfi, Véronique Harrivel, Florent Dumezy, Julien Guy, Mikael Roussel, Aguirre Mimoun, Pierre Fenaux, Nicolas Chapuis, Eric Solary, Dorothée Selimoglu-Buet, Orianne Wagner-Ballon, on behalf of the Groupe Francophone des Myélodysplasies (GFM)

**Affiliations:** 1 Département d’hématologie et immunologie biologiques, APHP, Hôpitaux universitaires Henri-Mondor, Créteil, France; 2 INSERM U955, Université Paris-Est, Créteil, France; 3 Laboratoire d’hématologie, Centre Hospitalo-universitaire Amiens-Picardie, Amiens, France; 4 Laboratoire d’hématologie, Centre Hospitalier Régional Universitaire de Lille, Lille, France; 5 Laboratoire d’hématologie, Centre Hospitalo-universitaire Dijon, Dijon, France; 6 Laboratoire d’hématologie, Centre Hospitalo-universitaire de Rennes, Rennes, France; 7 Laboratoire d’hématologie, Centre Hospitalier de la Côte Basque, Bayonne, France; 8 Service d’Hématologie clinique, Hôpital Saint-Louis, Paris, France; 9 Laboratoire d’hématologie, AP-HP, Hôpital Cochin, Paris, France; 10 INSERM UMR1170, Université Paris-Sud, Gustave Roussy, Villejuif, France

## Abstract

Peripheral blood monocytes include three subsets defined by CD14 and CD16 surface markers. An increase in the CD14^++^CD16^−^ classical monocyte fraction ≥ 94% of the total monocytes was proposed to rapidly and efficiently distinguish chronic myelomonocytic leukemia from reactive monocytosis. The robustness of this assay required a multicenter validation. The flow cytometry assay designed to quantify peripheral blood monocyte subsets was implemented by multiple diagnosis laboratories in France. A nationwide survey was performed to evaluate its performance. All the 48 French laboratories answered the questionnaire, revealing that 63% use this assay routinely. Central blind reanalysis of 329 cytometry files collected from five laboratories demonstrated an excellent correlation in classical monocyte fraction measurement (r = 0.93; *p* < 0.0001). The cutoff value of 94% classical monocytes being the critical readout for diagnosis, we then compared 115 patients with classical monocytes ≥ 94% and 214 patients with a fraction < 94% between initial analysis and reanalysis. An agreement was obtained in 311 files. Finally, an overt diagnosis, available for 86 files, confirmed a good sensitivity (93.6%) and specificity (89.7%). This survey demonstrates the robustness of the flow assay with limited variability of classical monocyte percentage between centers, validates the 94% cutoff value, and confirms its sensitivity and specificity.

## Introduction

The updated World Health Organization (WHO) classification for chronic myelomonocytic leukemia (CMML) diagnosis requires both the presence of persistent peripheral monocytosis ( ≥ 1 × 10^9^ /L) and monocytes accounting for ≥ 10% of the total white blood cell count^1,2^. Such a monocytosis can also be seen in various diseases, including chronic or acute infections, chronic inflammatory processes, and hematopoietic malignancies, more specifically myeloproliferative neoplasms^3,4^. CMML is characterized by a clonal hematopoiesis with abnormal myeloid differentiation, which is either dysplastic, leading to cytopenias, or exacerbated, leading to myeloproliferation, or both^5,6^. Dysplasia of one or more myeloid lineages might be observed, yet is not mandatory. If myelodysplasia is absent or minimal, an acquired clonal cytogenetic or molecular genetic abnormality should be detected for diagnosis statement^2^. Indeed, most CMMLs have somatic mutations, especially of *TET2*, *SRSF2*, *ASXL1* genes, and genes of the Ras pathway, although none of them are specific to the disease^7^. For all these reasons, diagnosis of CMML has hitherto remained difficult.

We found that accumulation of classical monocytes (cMo) CD14^++^CD16^−^ analyzed by flow cytometry, at the expense of intermediate monocytes (iMo) CD14^++^CD16^+^ and nonclassical (ncMo) CD14^−^/^low^CD16^+^, can be a powerful tool to diagnose CMML, regardless of mutational background, subtype, or dysplastic versus proliferative features. We showed that a relative accumulation of cMo ≥ 94% of total peripheral blood monocytes distinguishes CMML from any type of reactive monocytosis with high specificity (94.1%) and sensitivity (91.9%)^8^. This 94% threshold was subsequently validated in two other independent studies^9,10^, the latter highlighting the efficacy of this test to distinguish CMML from myeloproliferative neoplasms with associated monocytosis^10^. Moreover, the sensitivity of cMo accumulation was recently demonstrated to increase with the subtype, reaching 100% for CMML type 2^11^.

The flow cytometry-based monocyte subset analysis, hereafter referred to as “monocyte assay”, is now proposed as an additional diagnostic modality in CMML^12,13^. Since this assay has been largely adopted by diagnosis laboratories in France, we sought to assess its use throughout a nationwide survey and its performance through a multicenter evaluation.

## Methods

### Survey

A short questionnaire was sent by personalized emails between July and September 2016 to 48 French laboratories (mainly from university hospitals) performing routine flow cytometry diagnosis of myeloid neoplasms. Surveyed flow cytometrists were first asked whether they had implemented the “monocyte assay” in their daily routine practice. If not, they were queried if they would be willing to set it up within a short time, later, or most likely never. If they were using the “monocyte assay”, they were requested to provide several information, including the date and conditions in which the “monocyte assay” had been implemented, the antibody panel used, and the number of tests performed every month. Eventually, surveyed cytometrists were asked if they considered the “monocyte assay” useful for CMML diagnosis.

### Flow cytometry raw data

Five centers selected among those commonly using the assay were asked to send their raw data files as well as the corresponding cMo (CD14^++^CD16^−^) percentage evaluation in order to perform a centralized reanalysis. Briefly, whole-blood samples were stained with the following antibodies (all purchased from Beckman-Coulter, Brea, CA) as previously described^8^ and analyzed with a Navios Cytometer (Beckman-Coulter): CD14-PE or CD14-ECD or CD14-PC5.5 or CD14-APC or CD14-AA750 (clone RMO52); CD16-ECD or CD16-AA750 or CD16-PB (clone 3G8); CD2-FITC or CD2-PE or CD2-PC7 or CD2-AA700 (clone 39C1.5); CD56-PC5.5 or CD56-APC (clone N901); CD24-PE or CD24-PC5.5 (clone ALB9); CD45-KO (clone J33) for four centers; one center used CD7-AA700 (clone 8H8.1) instead of CD2 and CD33-PC5.5 (clone D3HL60.251) instead of CD24.

**Fig. 1:**
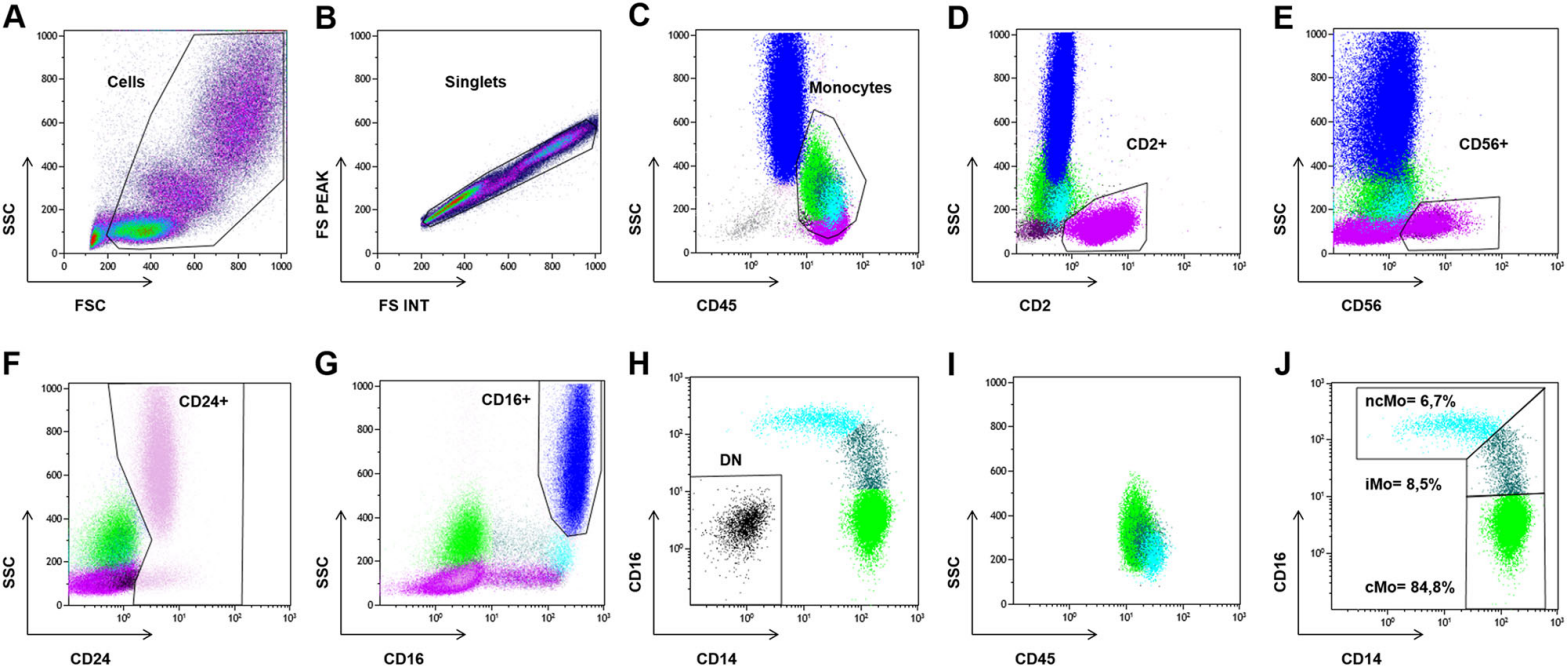
Exclusion gating strategy for monocyte subset determination. Example analysis of a raw data file provided by Center A. **a** Living cell selection on morphological parameters *(forward scatter FCS and side scatter SSC)*. **b** Singlets selection. **c** Monocytes rough selection as CD45^high^/SSC^int^ cells. **d** Exclusion of T cells selected as CD2^+^/SSC^low^ cells. **e** Exclusion of NK cells selected as CD56^+^/SSC^low/int^ cells (which overlap with ncMo CD14^−^/CD16^+^ subset on CD14/CD16 dot plot). **f** Exclusion of B cells selected as CD24^+^/SSC^low^ cells as well as both immature and mature granulocytes selected as CD24^+^/SSC^int to high^ cells. **g** Exclusion of residual mature granulocytes selected as CD16^high^ cells. **h** Exclusion of double-negative CD14^−^CD16^−^ cells. **i** Checking of the previous monocyte sub-population selection. **j** Separation of monocytes into CD14^++^/CD16^−^ (cMo, classical), CD14^++^/CD16^+^ (iMo, intermediate), and CD14^−^/CD16^+^ (ncMo, nonclassical) subsets

All the files received were analyzed in a blind fashion using Kaluza^®^ Software (Beckman-Coulter^®^) by two different operators, a skilled one (OWB) and a trainee one (ST) through a protocol adapted to each center and/or antibody panel used. Briefly, monocytes were roughly selected as CD45^high^/SSC^int^ cells among living cells and singlets (Fig. 1a–c). The other mature blood cells were then excluded, depending on the antibody panel used by the center that collected the data: T cells as CD2^+^/SSC^low^ (or CD7^+^/SSC^low^ cells), NK cells as CD56^+^/SSC^low/int^ cells (which overlap with ncMo subset on CD14/CD16 dot plot), B cells as CD24^+^/SSC^low^ cells, both immature and mature granulocytes as CD24^+^/SSC^int to high^ cells (or immature cells as CD33^+^/SSC^int to high^ cells), residual granulocytes expressing high levels of CD16, and the remaining CD14^−^CD16^−^ cells corresponding mainly to basophils and to NK cells which would have not previously been excluded (Fig. 1d–h). Afterward, the selection of the monocyte subpopulations was checked on a CD45/SCC dot plot (Fig. 1i), and if need be, the exclusion strategy was tweaked. Eventually, monocytes were separated on a CD14/CD16 scattergram into CD14^++^/CD16^−^ (cMo, classical), CD14^++^/CD16^+^ (iMo, intermediate), and CD14^−^/CD16^+^ (ncMo, nonclassical) subsets (Fig. 1j) as described^14–16^.

The percentages of cMo provided by the different centers were compared with cMo percentages determined by centralized analysis. Of note, the five selected centers used Navios instruments (Beckman-Coulter^®^, Miami, FL, USA).

### Biological and demographic parameters of the patients

The centers that did send raw data files were requested to provide biological and demographic data of the patients with an overt diagnosis.

### Statistical analysis

All data were collected using Excel software. We used GraphPad Prism software version 5.01 and MedCalc Statistical software version 12.7.5 (Ostend, Belgium) to perform correlation tests, Bland–Altman graphs, Mann–Whitney tests, and receiver-operating characteristic (ROC) curve.

### Ethics committee

This retrospective study was approved by the local Ethics Committee (IRB MONDOR).

## Results

### Most of the surveyed French centers use routinely the “monocyte assay”

**Fig. 2:**
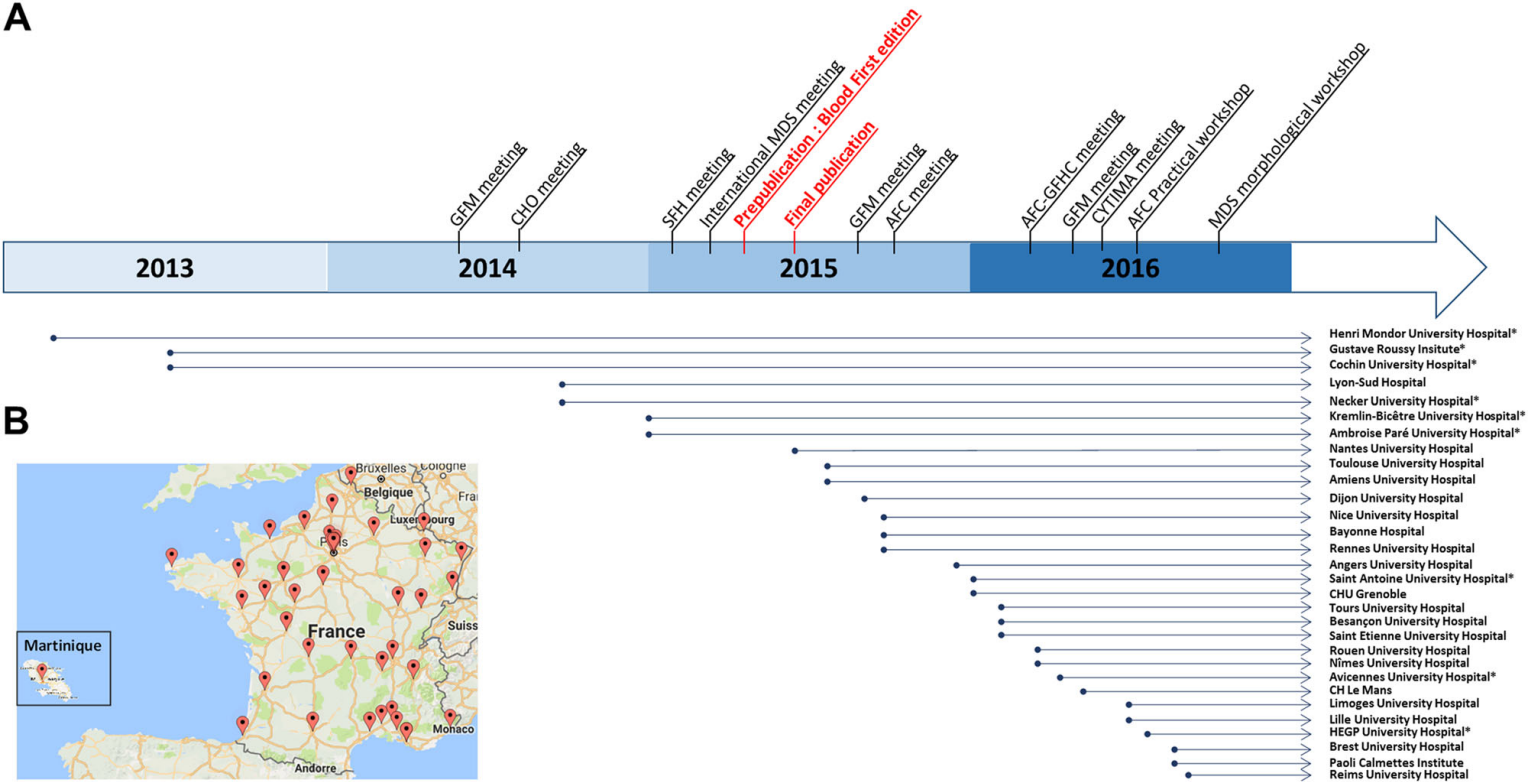
Current practices in France of the “monocyte assay”. **a** Chronological record of the “monocyte assay” implementation in the 30 French centers, in relation to the different oral communications on the subject in French or International meetings and the original publication in June 2015 (prepublished online as Blood First Edition paper in April 2015). CHO: “Club Hématopoïèse et Oncogenèse” (Hematopoiesis and Oncogenesis Club); SFH: “Société Française d’Hématologie” (French Society of Hematology); MDS: myelodysplastic syndrome; GFM: “Groupe Francophone des Myélodysplasies” (Myelodysplastic French-speaking Group); AFC: “Association Française de Cytométrie” (French Flow Cytometry Asociation); GFHC: “Groupe Francophone d’Hématologie Cellulaire” (French-speaking Group of Morphological Hematology); *Hospital centers in or around Paris. **b** Distribution of the 30 French centers that have implemented the “monocyte assay” (map obtained from Google Maps)

A short questionnaire was sent to the 48 French laboratories identified as performing routine flow cytometry diagnosis of myeloid neoplasms in order to assess the use and performance of the “monocyte assay”. We obtained a comprehensive reply to the survey with a 100% rate of response. Thirty of 48 centers (63%) use routinely the “monocyte assay”. Among the 18 centers that do not use this test, eight (44%) intend to develop it soon, the ten remaining centers not excluding a possible implementation. Apart from the three centers that participated in the original study^8^ (Henri Mondor University Hospital—Gustave Roussy Institute—Cochin University Hospital) and keep using the monocyte assay, 27 centers spontaneously implemented it, most often following the original publication and/or a meeting presentation (Fig. 2). It is noteworthy that, in 12 centers, the implementation was partly motivated by the request of the clinicians. Aside from the centers that participated in the original study, four centers, having listened to the first oral communications on the subject, set up the “monocyte assay” before the original publication in June 2015^8^.

Eighteen out of 27 (67%) centers use at least the published exclusion panel for non-monocyte mature blood cells^8^ (i.e., CD2 and CD56 for T lymphocytes and NK cells; CD24 for B lymphocytes and granulocytes). Ten centers use at least one exclusion marker, while two do not use any exclusion antibody. Among the 30 centers using the “monocyte assay”, nine centers perform 1–2 tests per month, fifteen 3–6 tests, three 6–10 tests, and three more than 10 tests per month. All the surveyed cytometrists consider the “monocyte assay” useful for CMML diagnosis.

### Excellent correlation of cMo percentages

In the second step, we sought to validate the routine use of the “monocyte assay” in centers that did not participate in the original study and had adapted the published technique. Having excluded the three centers which were part of the initial study, we selected five laboratories according to their antibody panel and their frequency of use, highlighting their keen interest in this test. Thus, we collected 329 useable flow cytometric raw data files provided by these centers, anonymized A to E. For each analyzed file, we collected the percentage of cMo provided by the center as well as the number of cMo events.

All received files were analyzed in a blind fashion by two different operators (skilled and trainee). The only 20 files showing discordant cMo percentages between these two analyses were reassessed in order to obtain a harmonized value. Given that no marker is common to the three sub-populations, positive gating of monocytes should be avoided. Indeed, markers classically used to select monocytes, such as CD14, CD64, CD33, or CD36, are expressed at a lower level by the ncMo subpopulation^15^, which would therefore be easily eliminated. Thus, the three monocyte subsets (cMo, iMo, and ncMo) were identified in total blood samples following the exclusion gating strategy originally published^8^, so as to eliminate the other mature circulating cells. All the five selected centers but one use the published exclusion antibody panel shown in Fig. 1, namely anti-CD2 and anti-CD56 antibodies to exclude NK cells, and anti-CD24 to exclude immature granulocytes. Center D uses distinct exclusion markers, including an anti-CD7 antibody to exclude NK cells and an anti-CD33 antibody to exclude immature granulocytes. Hence, an exclusion strategy settled for this specific antibody panel was used.

**Fig. 3:**
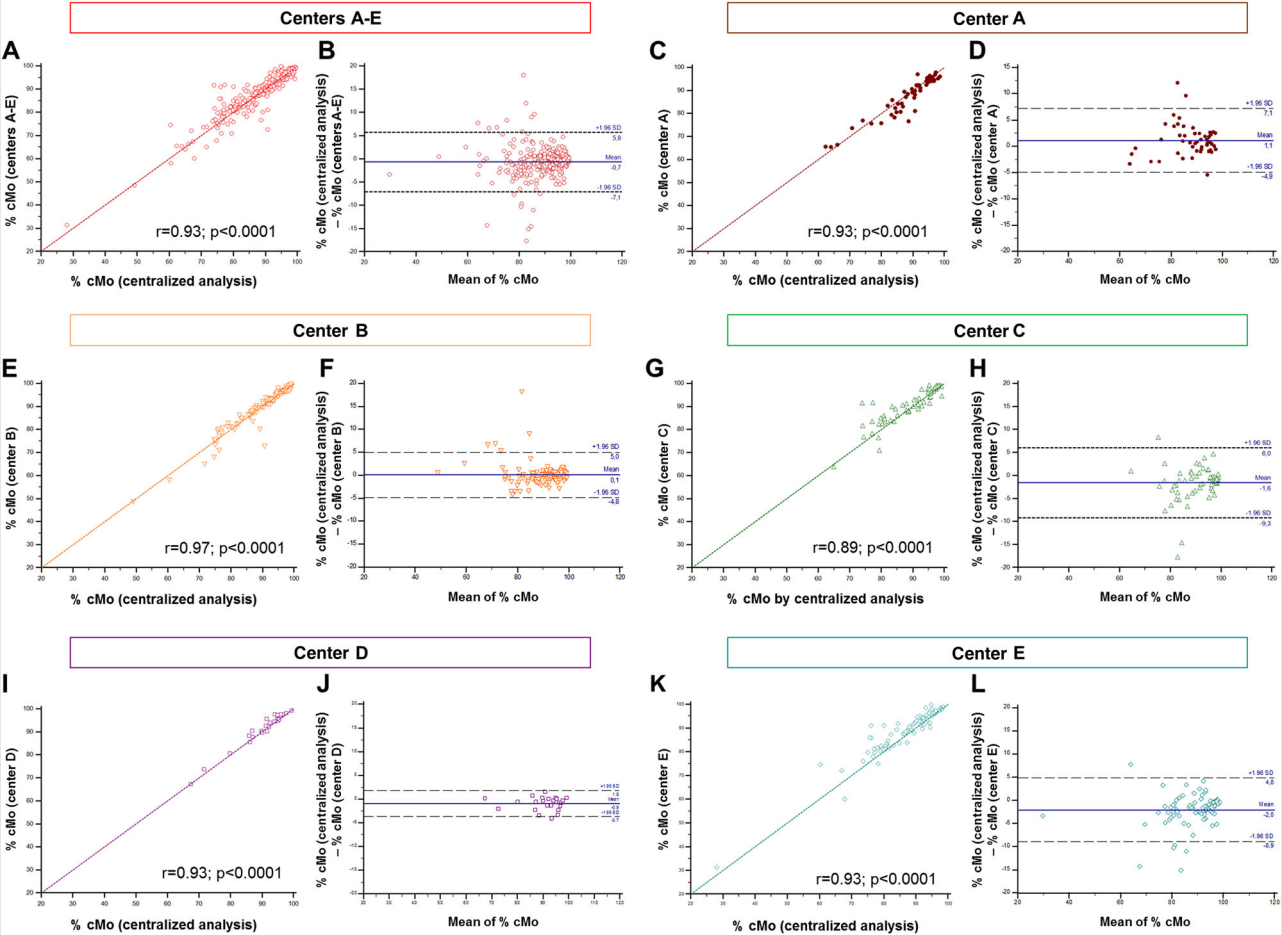
Correlation between the percentages of cMo supplied by five centers (**A**–**E**) and those determined by the centralized analysis for 329 flow cytometry raw data files. **a**, **c**, **e**, **g**, **i**, **k** Correlation between the percentages of cMo supplied by the five centers (A–E) and those determined by the centralized analysis. All percentages (%) were compared using a Pearson correlation test (correlation factor r is shown). **b**, **d**, **f**, **h**, **j**, **l** Bland–Altman plots showing putative bias between cMo percentages supplied by the five centers (A –E) and those determined by the centralized analysis

We compared the percentages of cMo arising from the agreement of the two blind analyses for the 329 FCM raw data files with those provided by the five centers. This comparison showed an excellent global significant correlation (center A to E: r = 0.93; *p* < 0.0001; Fig. 3a). The coefficients of correlation observed center by center ranged from 0.89 to 0.97 (center A: r = 0.93; B: r = 0.97; C: r = 0.89; D: r = 0.93; E: r = 0.93; *p* < 0.0001; Fig. 3c, e, g, i, and k).

Besides, no major bias was found between the percentages of cMo supplied by the five centers and those determined by the centralized analysis, as the mean of the differences was close to 0 (*Bland–Altman plot*, mean = –0.7; Fig. 3b). Among the five centers, center B displayed the strongest absence of bias (mean = 0.1; Fig. 3f). A very slight underestimation of the cMo percentages was observed with the values provided by center A (mean = 1.1; Fig. 3d). Conversely, we noted a trend to the overestimation of cMo percentages provided by centers C, D, and E (mean = −1.6, −0.9, and −2, respectively; Fig. 3h, j, and l). Altogether, correlation tests as well as bias studies did not show a noticeable difference between the percentages of cMo provided by the five centers and those determined by centralized analysis.

### Excellent agreement of cMo percentages related to the threshold value of 94% cMo

**Fig. 4:**
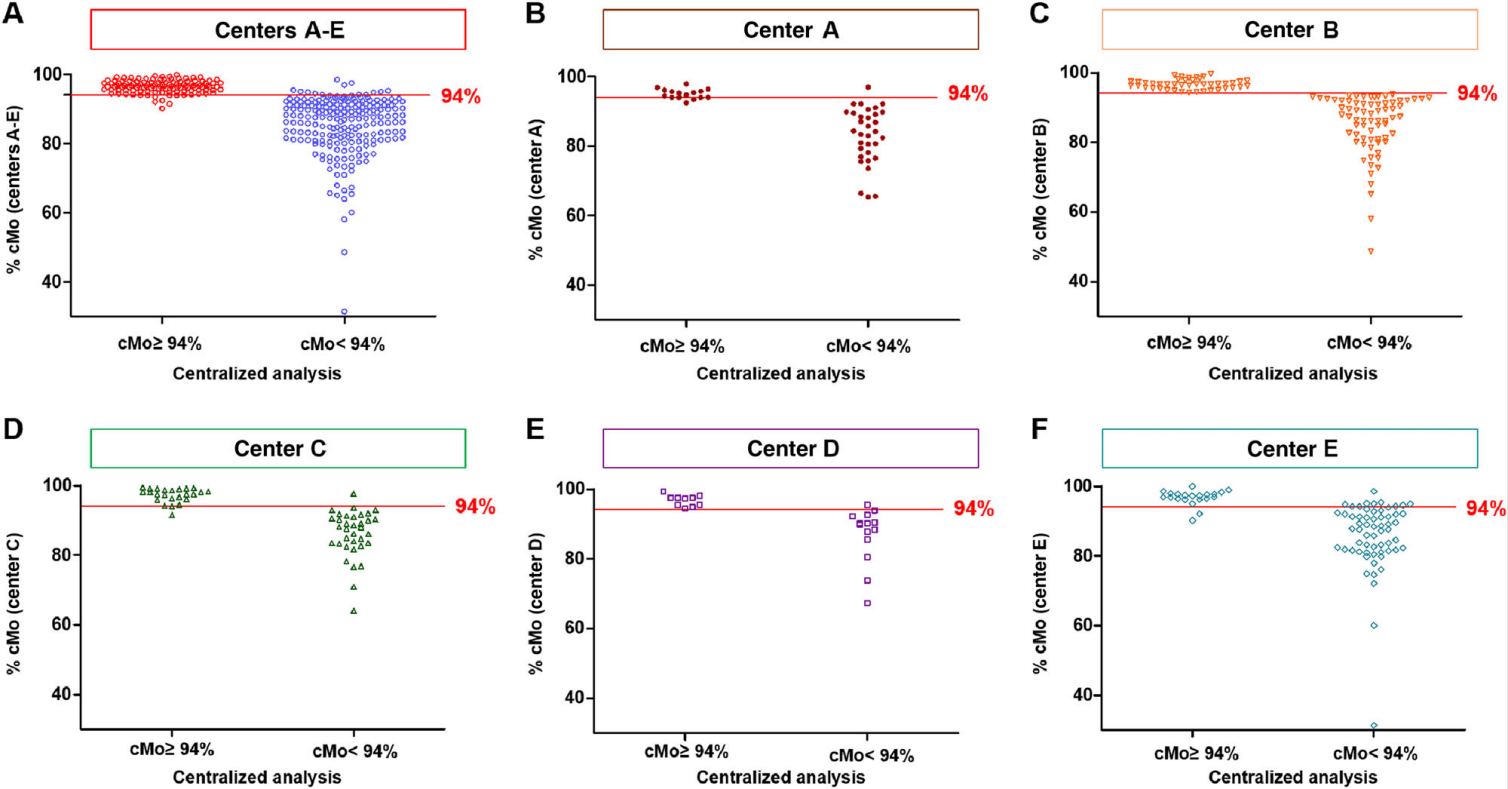
Agreement of cMo percentages supplied by five centers (**A**–**E**) and those determined by the centralized analysis for 329 flow cytometry raw data files related to the threshold value of 94% cMo. **a** Overall representation of all centers. **b**–**f** Representation of each center A to E

**Fig. 5:**
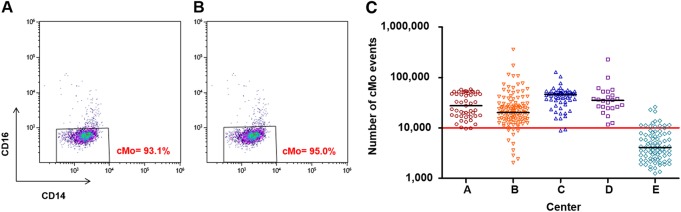
Significance of cMo event number acquired for “cMo gate” delineation. **a**, **b** Difficulty in drawing the “cMo gate” related to an insufficient number of cMo acquired in this example (only 3000 events), leading to a percentage below the value threshold of 94% (**a**) or above 94% (**b**). **c** Number of cMo events acquired by each center with the arbitrary threshold of 10,000 events represented by a red line. The median of cMo event number is indicated by the black bar for each center

As the threshold value of 94% cMo is the most relevant parameter in the “monocyte assay”, we compared patients for which the cMo percentage was ≥ 94% (i.e., suspected of being diagnosed as CMML)^8,11^. Both analyses (performed by each center and centralized) were in agreement (matching for 110 analyses/115 (95.7%), Fig. 4a). Conversely, of the 214 patients with a fraction of cMo < 94% by centralized analysis (i.e., not diagnosed as a CMML according to this parameter^8^), 201 (93.5%) displayed a percentage of cMo < 94% given by the center (Fig. 4a). Only one to two divergent cases were noted for center A to D (Fig. 4b–e), whereas Center E displayed ten underestimated cMo percentages and two overestimated ones (Fig. 4f). In most of these 18 divergent cases, an insufficient number of cMo had been acquired, which may have led to difficulties in delineating the “cMo gate” correctly (Fig. 5a,b). Hence, we decided arbitrarily to exclude all the files showing cMo event number below 10,000 (Fig. 5c), thereby improving both cMo percentage correlation (r = 0.94, *p* < 0.0001) and agreement for the 245 remaining files (total matching files: 95%).

### Multicenter validation of the 94% cMo threshold

Among the 245 files displaying more than 10,000 cMo events, we collected clinical data when available (demographic and hematological data, Table 1). We obtained 86 files associated to an overt diagnosis, namely 47 CMML according to the WHO 2017 criteria^2^, 23 reactive monocytosis, and 16 non-CMML malignancies. Among the 47 CMML patients, 32 (68%) were newly-diagnosed CMML. The 15 patients who had a pre-existing diagnosis received supportive care (e.g., blood transfusion and erythropoiesis-stimulating agents) or cytoreductive drugs, but none of them were treated with a hypomethylating agent.

**Table 1:** Demographic and hematological data parameters in CMML patients, patients with reactive monocytosis, and patients with non-CMML malignancies

	Total CMML	CMML-0	CMML-1	CMML-2	Reactive monocytosis	Non-CMML malignancies
Patients, *n* (%)	47 (100)	14 (30)	26 (55)	7 (15)	23 (100)	16 (100)
Age, years	75 ± 13	78 ± 8	74 ± 15	73 ± 13	63 ± 23	76 ± 11
M/F (sex ratio)	30/17 (1.8)	8/6 (1.3)	17/9 (1.9)	5/2 (2.5)	17/6 (2.8)	9/7 (1.3)
CBC, *n* (%)	40 (85)	13 (93)	23 (88)	6 (86)	18 (78)	14 (88)
Hemoglobin, g/dL	11.5 ± 2.1	12.0 ± 2.3	11.5 ± 2.1	10.6 ± 1.7	11.8 ± 2.4	10.2 ± 1.9
Platelets, x10^9^/L	156 ± 115	144 ± 128	167 ± 120	138 ± 64	315 ± 157	338 ± 258
WBC, x10^9^/L	18.9 ± 26.8	15.3 ± 13.6	15.8 ± 16.4	38 ± 60	27.6 ± 47.2	20.9 ± 18.7
Neutrophils, x10^9^/L	10.5 ± 14.9	9.2 ± 7.7	8.8 ± 9.8	19.7 ± 33	17.3 ± 26.4	13.6 ± 14.3
Monocytes, x10^9^/L	3.9 ± 3.2	3.2 ± 2.6	3.7 ± 3.11	5.7 ± 4.4	4.3 ± 10.3	2.5 ± 2.0
Monocytes, %	26.0 ± 10.4	23.2 ± 7.5	27.6 ± 11.3	25.4 ± 11.2	15.1 ± 7.7	14.2 ± 8.6

All parameters are mean ± standard deviation. CMML patients were subdivided into three groups, CMML-0, CMML-1, and CMML-2, according to the WHO classification^2^. Non-CMML malignancy patients were six myelodysplastic syndromes, two chronic myeloid leukemias, two myelodysplastic/myeloproliferative neoplasms with ring sideroblasts and thrombocytosis, one essential thrombocythemia, one non-Hodgkin lymphoma, two malignant blood diseases with JAK2 mutation, and eventually two non-CMML malignancies unspecified by centers

The average age of CMML patients was 75 ± 13 years with male predominance (sex ratio *=* 1.8). White blood cell (WBC) counts were variable with a mean of 18.9 ± 26.8 × 10^9^/L (up to 161 × 10^9^/L), and the absolute monocyte count was 3.9 ± 3.2 × 10^9^/L with a monocyte percentage of 26.0 ± 10.4%. These patients displayed both mild anemia (mean hemoglobin: 11.5 ± 2.1 g/dL) and thrombocytopenia (mean platelets: 156 ± 115 × 10^9^/L). Patients with reactive monocytosis were younger than CMML patients (63 ± 23 years), with also a majority of males (sex ratio = 2.8). The mean WBC count was higher (27.6 ± 47.2 × 10^9^/L) related to the increase of neutrophils in these reactive contexts, and the mean absolute monocyte count was 4.3 ± 10.3 × 10^9^/L. Patients with non-CMML malignancies had age close to that of CMML patients (76 ± 11 years), and presented with a mean WBC count of 20.9 ± 18.7 × 10^9^/L with a mean absolute monocyte count of 2.5 ± 2 × 10^9^/L (Table 1).

**Fig. 6:**
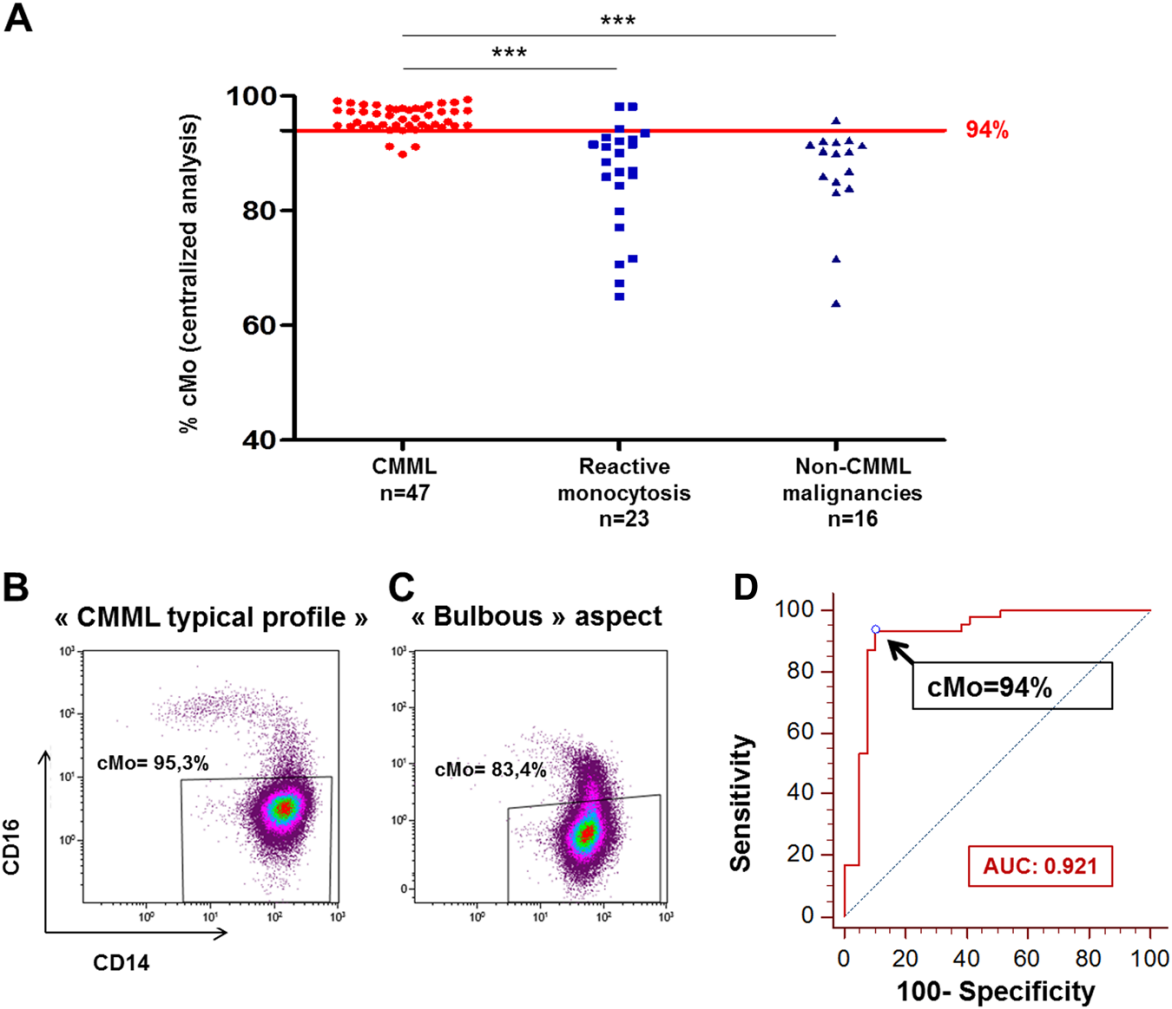
Sensitivity and specificity of the “monocyte assay”. **a** cMo percentages determined by centralized analysis for CMML patients (median: 96.1% [interquartile range: 94.9–97.8]), patients with reactive monocytosis (median: 88.5% [interquartile range: 79.9–92.5]), and patients with non-CMML malignancies (median: 90.2% [interquartile range: 84.3–91.8]). ***p *<* 0.0001 (Mann–Whitney test). **b**–**c** Examples for cMo percentages for two CMML patients, one CMML typical profile with cMo > 94% (**b**) and one “bulbous aspect” observed when CMML is associated with an inflammatory state (**c**). **d** Receiver-operating curve (ROC) establishing a 94% cMo cutoff value

To determine the sensitivity and specificity of the “monocyte assay” from these data, we used the cMo percentages obtained from the centralized analysis, owing to the 18 mismatches with the percentages supplied by the centers previously described. Accumulation of cMo ≥ 94% was observed in 44 of the 47 CMML, indicating a sensitivity of 93.6% (Fig. 6a). The three false negatives showed the characteristic “bulbous” aspect observed when CMML is associated with an inflammatory state^11^. This easily recognized profile is due to the disappearance of ncMo (CD14^−/low^CD16^+^) subset combined with the increase of iMo (CD14^++^CD16^+^) subset (Fig. 6b,c). Indeed, in two of these three patients, the bulbous aspect could be related with an inflammatory state, either a pericarditis or a colon cancer with, in both situations, an elevated C-reactive protein (mean: 60.5 mg/L). Clinical information regarding the third case was missing. Hence, the “corrected” sensitivity of the “monocyte assay” reached 100%.

A specificity of 89.7% was calculated as 35 of the 39 “non-CMML” patients displayed cMo < 94% determined by centralized analysis. The four false positives consisted of three diagnoses of reactive monocytosis and one MDS. Interestingly, using these data provided by these five different centers, we established a receiver-operating curve (ROC) and obtained yet again a 94% cutoff value of cMo with an area under the ROC curve (AUC) of 0.921 (Fig. 6d).

## Discussion

Peripheral monocytosis is a very common finding in routine laboratories. Since CMML should be suspected based on this sole arbitrary criterion, bone marrow examination is required for dysplasia assessment, blast quantification, and cytogenetic evaluation if no reactive causes can explain the sustained elevated absolute monocyte count^2^. Even though recurrent cytogenetic aberrations are found in 30–40% of CMML patients^17^, detection of such a clonal abnormality can help confirm diagnosis, especially in the absence of significant dysplasia^2^. About 90% of CMML patients have somatic mutations including *TET2*, *SRSF2*, and *ASXL1* mutations^7,18–20^ with an average of 14 per patient^21^, yet none of them are specific to the disease. Additional mutations in genes of the Ras pathway are frequently detected in the proliferative form of the disease. Since some of these mutations can be detected in otherwise healthy older patients^22,23^, detection of such mutations may be not sufficient to confirm a neoplastic origin of the monocytosis;^24^ hence, there is a need to develop a diagnostic tool available as of peripheral blood examination. We showed 3 years ago that the “monocyte assay” was very effective in distinguishing CMML from reactive monocytosis. As an increasing number of laboratories regularly request our advice for raw data file interpretation, we thought of carrying out a survey focused on the practices of this test in France. This study reveals a strong adhesion of the French laboratories to the “monocyte assay” with 30 user centers. The number of tests performed in the different laboratories are heterogeneous, half of them using it three to six times a month. This low frequency reflects a targeted use of the “monocyte assay” in a context of CMML suspicion, and not a systematic use of this test for monocytosis management. Indeed, this mutiparameter flow cytometry assay may hardly be performed for each peripheral blood sample displaying an absolute monocyte count ≥ 1 × 10^9^/L. In this regard, we proposed a new application of the Hematoflow™ solution^25^ (Beckman-Coulter, Brea, CA) which provides white blood cell (WBC) differentials by flow cytometry^26^ and allows the quantification of the CD16-negative monocyte fraction at the very same time. Hence, it provides a useful approximation of the cMo percentage and may easily detect samples that are suspected of being CMML and thus require further exploration by the “monocyte assay”.

This original study published in 2015 included a multicenter validation of this test carried out by three laboratories with a standardized protocol. We sought here to validate the use of the “monocyte assay” by independent centers which were not part of the initial study and without prior standardization. The only criterion for center selection was the total number of tests performed and the antibody panel exclusion design, which should contain markers for both NK cells and immature granulocytes as these latter populations may overlap with ncMo and lead to a cMo percentage underestimation in the case of flawed exclusion. Thus, laboratories that were in training or that had just implemented the “monocyte assay” were not included. The type of flow cytometer was not a selection criterion since both Becton-Dickinson (LSR II) and Beckman-Coulter (Navios) instruments were used in our previous studies^8,11^. By chance, the five selected centers all used Navios instruments (Beckman-Coulter).

We received 329 files flow cytometry raw data with the corresponding cMo percentages and decided to duplicate their blind centralized analysis by a trainee operator and a skilled one in order to evaluate the easiness of the gating strategy once it has been set up. Both analyses were extremely correlated with very few discordant files. The cMo percentages arising from this agreement showed a very good global correlation with the cMo percentages provided by centers. Furthermore, the absence of major bias was a key input of this study, as the “monocyte assay” interpretation is related to a cutoff value.

Hence, we decided to focus on the agreement of the cMo percentages below or above the 94% threshold. Only 18 out of the 329 files were discordant. Most of them were due to an insufficient number of cMo events acquired. Indeed, in this case, the separation of the cMo and the iMo populations may be tricky. We highlight here the usefulness of the density plot representation for the “cMo gate” delineation. Further exchange with the center E that provided the highest number of files with cMo events below 10,000 unveiled that only 50 µl of total blood had been used instead of 200 µl as recommended. These results highlight the importance of the pre-analytical conditions for the “monocyte assay” implementation.

An accumulation of cMo ≥ 94% was observed in 44 of the 47 CMML, indicating a sensitivity of 93.6% according to previous results. The three false negatives showed a specific abnormal profile, drawing a “bulbous aspect” on the CD14/CD16 dot plot, related to an increase of the iMo subset combined to the disappearance of the ncMo population, which has been recently described in CMML patients with an inflammatory state^11^. Concurrent cases of autoimmune diseases and/or systemic inflammatory syndromes have been reported in 20% of CMML patients^27^. In such cases, an increase of the iMo subset can lead to an underestimation of the relative cMo percentage, which drops below the 94% threshold^11^. Considering the three false-negative cases as genuine CMML, we obtained a “corrected” sensitivity of 100%. A specificity of 89.7% was calculated with five false positives (i.e., non-CMML patients with cMo ≥ 94%) consisting of four reactive monocytosis and one MDS. Interestingly, a relative accumulation of cMo ≥ 94%, i.e., “a CMML-like signature” was described in roughly one-third of myelodysplastic syndromes at diagnosis^9,11^, a fraction of them being able to evolve into a genuine CMML^11^. These data highlighted the significance of the monocyte component in MDS reported by different studies. MDS with a relative monocytosis^28^, i.e., monocytes accounting for 10% of peripheral leukocytosis yet below 1 × 10^9^/L or MDS with marrow monocytosis^29^, display a similar clinicopathologic and mutational profile to classical CMML^29,30^. Further investigations are needed to characterize these early stages of CMML which do not currently fulfill the WHO criterion of monocytosis ≥ 1 × 10^9^/L.

Eventually, this new independent cohort of patients allowed us to challenge the previous 94% threshold established in 2015^8^. The AUROC test performed using these new data led yet again to a 94% cutoff value of cMo, highlighting the robustness of this threshold.

The simplicity of the “monocyte assay”, performed on whole-blood sample with a limited number of antibodies, the antibody exclusion panel not being imposed, and the ease of interpretation confirmed by the centralized review have favored the implementation of this phenotypic test in 30 laboratories in France. Our study confirms the successful multicenter use of this flow cytometry test, provided a minimum of 10,000 events were analyzed. This analysis demonstrates a low variability of cMo percentage quantification and validates the previously proposed 94% cutoff value, defining a robust, sensitive, and specific assay. Compared with genetic analyses, the simplicity of this flow cytometry approach may be likely increasingly widespread^31^, pending the demonstration of its clinical benefit that requires a prospective, international multicenter study.
